# Skeletal Muscle Mitochondrial Respiration and Exercise Intolerance in Patients With Heart Failure With Preserved Ejection Fraction

**DOI:** 10.1001/jamacardio.2023.0957

**Published:** 2023-05-10

**Authors:** Lina Scandalis, Dalane W. Kitzman, Barbara J. Nicklas, Mary Lyles, Peter Brubaker, M. Benjamin Nelson, Michelle Gordon, John Stone, Jaclyn Bergstrom, P. Darrell Neufer, Erich Gnaiger, Anthony J. A. Molina

**Affiliations:** 1 Division of Geriatrics, Gerontology, and Palliative Care, UC San Diego School of Medicine, University of California, San Diego; 2 Cardiovascular Medicine Section, Department of Internal Medicine, Wake Forest School of Medicine, Winston-Salem, North Carolina; 3 Section on Gerontology and Geriatric Medicine, Wake Forest School of Medicine, Winston-Salem, North Carolina; 4 Department of Health and Exercise Science at Wake Forest University, Winston-Salem, North Carolina; 5 Department of Physiology, Brody School of Medicine, East Carolina University, Greenville, North Carolina; 6 Oroboros Instruments, Innsbruck, Austria

## Abstract

**Importance:**

The pathophysiology of exercise intolerance in patients with heart failure with preserved ejection fraction (HFpEF) remains incompletely understood. Multiple lines of evidence suggest that abnormal skeletal muscle metabolism is a key contributor, but the mechanisms underlying metabolic dysfunction remain unresolved.

**Objective:**

To evaluate the associations of skeletal muscle mitochondrial function using respirometric analysis of biopsied muscle fiber bundles from patients with HFpEF with exercise performance.

**Design, Setting, and Participants:**

In this cross-sectional study, muscle fiber bundles prepared from fresh vastus lateralis biopsies were analyzed by high-resolution respirometry to provide detailed analyses of mitochondrial oxidative phosphorylation, including maximal capacity and the individual contributions of complex I–linked and complex II-linked respiration. These bioenergetic data were compared between patients with stable chronic HFpEF older than 60 years and age-matched healthy control (HC) participants and analyzed for intergroup differences and associations with exercise performance. All participants were treated at a university referral center, were clinically stable, and were not undergoing regular exercise or diet programs. Data were collected from March 2016 to December 2017, and data were analyzed from November 2020 to May 2021.

**Main Outcomes and Measures:**

Skeletal muscle mitochondrial function, including maximal capacity and respiration linked to complex I and complex II. Exercise performance was assessed by peak exercise oxygen consumption, 6-minute walk distance, and the Short Physical Performance Battery.

**Results:**

Of 72 included patients, 50 (69%) were women, and the mean (SD) age was 69.6 (6.1) years. Skeletal muscle mitochondrial function measures were all markedly lower in skeletal muscle fibers obtained from patients with HFpEF compared with HCs, even when adjusting for age, sex, and body mass index. Maximal capacity was strongly and significantly correlated with peak exercise oxygen consumption (*R* = 0.69; *P* < .001), 6-minute walk distance (*R* = 0.70; *P* < .001), and Short Physical Performance Battery score (*R* = 0.46; *P* < .001).

**Conclusions and Relevance:**

In this study, patients with HFpEF had marked abnormalities in skeletal muscle mitochondrial function. Severely reduced maximal capacity and complex I–linked and complex II–linked respiration were associated with exercise intolerance and represent promising therapeutic targets.

## Key Points

**Question:**

Does mitochondrial dysfunction underlie altered skeletal muscle metabolism and exercise intolerance in patients with heart failure with preserved ejection fraction (HFpEF)?

**Findings:**

In this cross-sectional study including 27 patients older than 60 years with HFpEF and 45 healthy age-matched controls, high-resolution respirometry of vastus lateralis muscles from patients with HFpEF revealed markedly reduced bioenergetic capacity associated with peak exercise oxygen consumption and exercise performance (6-minute walk distance, Short Physical Performance Battery, and leg strength).

**Meaning:**

In this study, detailed analysis of mitochondrial function provided evidence that skeletal muscle mitochondrial dysfunction can play a role in HFpEF exercise intolerance, which may impact the development of therapeutic strategies that target mitochondrial dysfunction in patients with HFpEF.

## Teaser

This cross-sectional study evaluates the associations of skeletal muscle mitochondrial function using respirometric analysis of biopsied muscle fiber bundles from patients with heart failure with preserved ejection fraction (HFpEF) with exercise performance.

## Introduction

Heart failure (HF) with preserved ejection fraction (HFpEF) is the most prevalent form of HF, particularly among older adults and women.^1,2^ As our population continues to get older, the prevalence of HFpEF is increasing,^3,4^ as are risk factors such as obesity, diabetes, and hypertension.^5,6^

The primary clinical manifestation of chronic stable HFpEF is severe exercise intolerance, which is associated with impaired quality of life, and can be measured objectively and reproducibly as reduced peak exercise oxygen uptake (peak VO_2_).^7,8,9,10^ However, the pathophysiology of exercise intolerance is incompletely understood, and there are few effective therapies. Multiple lines of evidence indicate that in addition to underlying cardiac dysfunction, noncardiac factors contribute to exercise intolerance in HFpEF.^9,11,12^ Our group and others reported that reduced cardiac output accounts for only approximately 50% of the reduced peak VO_2_ in patients with HFpEF,^9,13^ supporting a role for peripheral factors. Endurance exercise training significantly improves peak VO_2_ in clinically stable older patients with HFpEF, with most of the improvement mediated by noncardiac factors, such as skeletal muscle function.^14,15^ Altogether, these studies suggest that skeletal muscle alterations significantly contribute to exercise intolerance in patients with HFpEF.

Several lines of evidence indicate that skeletal muscle metabolism is impaired in patients with HFpEF.^12^ While patients with HFpEF have a lower lean mass, the increase in VO_2_ during exercise relative to lean mass is lower in those with HFpEF compared with healthy controls (HCs), suggesting intrinsic metabolic differences.^16^ We have reported that older patients with HFpEF have abnormal skeletal muscle oxygen utilization that is associated with severely reduced peak VO_2_.^16^ Using magnetic resonance spectroscopy, Weiss et al^17^ reported reduced skeletal muscle oxidative metabolism and its relation to muscle fatigue. Further, Dhakal et al,^13^ using hemodynamic monitoring during exercise, showed that oxygen extraction was significantly reduced in HFpEF and is a major contributor to reduced peak VO_2_.

Examination of skeletal muscle biopsies has shown that patients with HFpEF have a decreased number of type I oxidative fibers.^18^ Patients with HFpEF have lower vastus lateralis mitochondrial content and oxidative capacity as reported by citrate synthase activity and the expression of mitochondrial structural proteins.^19^ The significant associations of these parameters with measures of exercise capacity support that these deficits may contribute to severely reduced exercise capacity. Additionally, we observed that the mitochondrial fusion regulator, mitofusin 2 (Mfn2), is significantly decreased in HFpEF skeletal muscle and may also contribute to exercise intolerance. Importantly, these mitochondrial parameters were directly related to peak VO_2_ and 6-minute walk distance.

Despite evidence suggesting multifaceted skeletal muscle mitochondrial impairments, respirometric analyses of skeletal muscle tissue, the criterion-standard assessment of mitochondrial function, in the context of HFpEF are lacking. Using a rat model of HFpEF, Bowen et al^20^ reported impaired mitochondrial respiration that was ameliorated with exercise training. More recently, skeletal muscle maximal mitochondrial respiration was found to be 40% to 55% lower in a postmenopausal rat model of HFpEF compared with controls.^21^ This was accompanied by a 15% to 30% decrease in specific force generation, suggesting a role for mitochondrial abnormalities in skeletal myopathy.

To elucidate the role of mitochondria in impaired skeletal muscle metabolism in patients with HFpEF, the study presented here used high-resolution respirometry of freshly obtained skeletal muscle specimens to provide detailed analyses of mitochondrial function, including precise assessments of the maximal capacity of the electron transfer system and the individual contributions of complex I–linked and complex II–linked respiration. These bioenergetic data were then associated with key measures of exercise performance across HFpEF and age-matched HC participants.

## Methods

### Participants

Participants with HFpEF were selected based on detailed inclusion criteria, as described previously^7,9,15,22,23,24^ and in accord with the 2013 American College of Cardiology/American Heart Association recommendations current at the time of study design.^25^ HFpEF was defined as symptoms and signs of HF and preserved resting left ventricular systolic function (50% or greater), left ventricular diastolic dysfunction of grade 1 or higher, and body mass index (BMI; calculated as weight in kilograms divided by height in meters squared) of 28 or greater. HF signs and symptoms were confirmed by a board-certified cardiologist and met the criteria of the National Health and Nutrition Examination Survey HF clinical score of 3 or higher and the criteria of Rich et al.^26,27^ Exclusions included significant ischemic or valvular heart disease, pulmonary disease, anemia, or other disorder that could explain the patients’ symptoms.^7,9,23^ Given the strong age dependence of HFpEF, patients and controls were 60 years and older at study entry. Participants in this study were drawn from a parent clinical trial of patients with HFpEF. The results of that trial, including a detailed description of the study participants, have been published.^28^

Age-matched, healthy, sedentary persons were recruited to serve as HCs. Potential HCs were excluded if they had chronic medical illness, were taking chronic medications other than preventive low-dose aspirin, had abnormal findings on physical examination (including blood pressure of 140/90 mm Hg or higher), had abnormal results on the screening tests (including electrocardiography, exercise echocardiography, and spirometry), or regularly undertook vigorous exercise.^16,29^ The protocol for this study was approved by the Wake Forest School of Medicine institutional review board, and all participants provided written informed consent.

### Exercise Performance

Cardiopulmonary exercise testing was performed on a treadmill using the modified Naughton protocol for patients with HFpEF and using the modified Bruce protocol for HCs, as previously described.^7,24,30^ Expired gas analysis was conducted using a commercially available system (CPX-2000 and Ultima; MedGraphics), calibrated before each test with a standard gas of known concentration and volume. Breath-by-breath gas exchange data were measured continuously during exercise and averaged every 15 seconds, and peak values were averaged from the last two 15-second intervals during peak exercise.

A 6-minute walk test was performed using the method of Guyatt et al.^31^ The Short Physical Performance Battery (SPPB) consists of a usual gait speed test, a usual gait speed test using a narrow course (20 cm), 5 repeated chair stands, and a 30-second standing balance tests (side-by-side, semitandem, and full tandem).^32,33^ Each component is scored on a scale of 0 to 4 for a total score of 0 to 12, with a higher score indicating better physical function.

Peak upper leg strength (in newton meters) was measured on a dynamometer (Biodex Medical Systems) at 60° per second, with the participant seated and the hips and knees flexed at 90°. To stabilize the hip joint and the trunk, participants were secured with straps at the chest, hip, and thigh. Seat height and depth, and the position of the lever arm ankle pad were adjusted to accommodate each participant. Participants were asked to extend the knee and push as hard as possible against the ankle pad. Strength of the right and left legs recorded as peak torque was used for analyses.

### Skeletal Muscle Biopsy

Vastus lateralis biopsies were performed in the morning after an overnight fast, as previously described.^18,34,35^ Participants were asked to refrain from taking aspirin, nonsteroidal anti-inflammatory drugs, and other compounds that may affect bleeding, platelets, or bruising for the week prior to the biopsy and to refrain from strenuous activity for 36 hours prior to biopsy. Muscle was obtained from the vastus lateralis by percutaneous needle biopsy using a University College Hospital needle under local anesthesia with 1% lidocaine.^36^ Visible blood and connective tissue were removed from muscle specimens, and portions for mitochondrial analyses were immediately analyzed by respirometry.

### High-Resolution Respirometry of Permeabilized Skeletal Muscle Fibers

Mitochondrial oxidative phosphorylation can be evaluated by measuring the rate of oxygen consumption in cells and tissues.^37,38,39^ High-resolution respirometry of permeabilized skeletal muscle fiber bundles was performed following a protocol in which substrates and inhibitors are sequentially added to measure oxygen flux mediated by mitochondrial complexes I and II respiration, as well as maximal uncoupled respiration maximal capacity. Together, these primary outcomes (complex I respiration, complexes I and II respiration, and maximal capacity) report on the maximal bioenergetic capacity of the electron transport system and the contributions of the 2 major electron transport chain entry points to this capacity. Following previously published protocols,^40^ approximately 2.5 mg (wet weight) of tissue were loaded into each of 2 chambers of an Oroboros Oxygraph-2k (Oroboros), and steady-state rate of respiration measurements were obtained after every substrate addition and expressed as picomoles per second per milligram of tissue.^41^ High-resolution oxygen flux measurements were measured in 2-mL buffer Z containing 20mM of creatine and 25μM of blebbistatin to inhibit contraction.^41^ This injection protocol was completed as follows: 2mM of malate, 4mM of adenosine diphosphate, 20mM of pyruvate, 10mM of glutamate (complex I substrates), 10mM of succinate (complex II substrates), 10μM of cytochrome *c* to test for mitochondrial membrane integrity, 2 additions of 0.25μM of carbonyl cyanide p-trifluoro methoxyphenylhydrazone (FCCP) followed by a titration of 0.5μM of FCCP to obtain maximal electron transfer capacity, 0.5μM of rotenone, and 5μM of antimycin A. Each sample was run in duplicate, and all data were normalized to measured muscle fiber bundle wet weight. All assays were performed under a high initial oxygen concentration (350uM to 400uM) in the O2K chamber (Oroboros Instruments).

### Statistical Analysis

Shapiro-Wilk tests were performed to check for normal distribution of all variables. Log transformations were performed for parameters with nonnormal distribution. Intergroup (HFpEF vs HC) comparisons of participant characteristics were made by independent-samples *t* tests and χ^2^ tests. Intergroup differences in bioenergetics parameters were compared using independent-samples *t* tests; additionally, to account for the differences in sex, BMI, and age, adjustments for these variables were made using analysis of covariance. Intergroup in-exercise and physical function measures were assessed using independent-samples *t* tests as well as adjustment for sex using analysis of covariance. Pearson correlation coefficients were assessed between all variables, both raw and normalized values, and partial correlations adjusted for age, sex, and BMI were also assessed. Significance between groups was defined as *P* < .05, and all *P* values were 2-tailed. Analyses were performed using SPSS software version 26 (IBM).

## Results

### Participant Characteristics

Of 72 included patients, 50 (69%) were women, and the mean (SD) age was 69.6 (6.1) years. A total of 27 patients with HFpEF and 45 age-matched HCs were included. Patients with HFpEF had characteristics typical of population-based studies of chronic, stable HFpEF with New York Heart Association class II to III symptoms with increased left ventricular mass and abnormal Doppler left ventricular diastolic function compared with HCs and with typical comorbidities (27 [100%] with hypertension and 10 [37%] with diabetes), and 25 [93%] were taking diuretics (Table 1). Patients with HFpEF and HCs were well matched for age; however, there were more women in the HFpEF group. Body mass, fat mass, percentage body fat, and BMI were higher in those with HFpEF compared with HCs, in accord with observations in multiple population-based studies and trials that have reported significantly higher BMI in patients with HFpEF compared with the general population.^42,43,44,45,46^

**Table 1.** Characteristics of Patients With Heart Failure With Preserved Ejection Fraction (HFpEF) and Age-Matched Healthy Controls (HCs)

Characteristic	No. (%)		*P* value
	Patients with HFpEF (n = 27)	HCs (n = 45)	
Age, mean (SD), y	68.4 (5.8)	70.2 (6.2)	.22
Gender			
Men	4 (15)	9 (20)	.39
Women	23 (85)	36 (80)	
Height, mean (SD), cm	163.8 (8.3)	165.8 (9.7)	.38
Weight, mean (SD), kg	104.9 (20)	74.1 (14.3)	<.001
Body mass index, mean (SD)^a^	38.9 (6.2)	26.8 (3.7)	<.001
Body surface area, mean (SD), m^2^	2.08 (0.22)	1.82 (0.21)	<.001
Total fat mass, mean (SD), kg	51.8 (12.3)	29.6 (7.7)	<.001
Total lean mass, mean (SD), kg	54.4 (10.1)	43.4 (9.7)	<.001
Body fat, mean (SD), %	49 (5)	39 (7)	<.001
Systolic blood pressure, mean (SD), mm Hg	144.9 (20.5)	121.6 (11)	<.001
Diastolic blood pressure, mean (SD), mm Hg	75 (11)	69 (9)	.01
Ejection fraction, mean (SD), %	61 (4)	59 (4)	.45
Left ventricular mass, mean (SD), g	205.4 (45.8)	156.8 (33.8)	<.001
Left ventricular mass index, mean (SD)	98.3 (18.8)	83.8 (4.4)	.02
Left atrial diameter, mean (SD), cm	3.9 (0.4)	3.7 (0.5)	.12
E/A ratio, mean (SD)	0.97 (0.32)	0.86 (0.23)	.21
e′ septal, mean (SD), cm/s	6.4 (1.9)	7.8 (1.5)	.02
E/e′ ratio, mean (SD)	13.3 (6.4)	9.4 (2.8)^b^	.031
N-terminal pro–brain natriuretic peptide, median (IQR), pg/mL	86 (82-145)^c^	NA	NA
Diastolic filling pattern			
Normal	1 (4)	3 (20)	.03
Impaired	24 (89)	8 (53)	
Pseudonormal	2 (7)	4 (27)	
Restrictive	0	0	
History of hypertension	27 (100)	NA	NA
History of atrial fibrillation	2 (7)	NA	NA
History of coronary heart disease	5 (19)	NA	NA
Diabetes	10 (37)	NA	NA
New York Heart Association HF class			
I	1 (4)	NA	NA
II	8 (31)	NA	NA
III	12 (46)	NA	NA
IV	5 (19)	NA	NA
Medications			
Diuretics	25 (93)	NA	NA
Angiotensin-II receptor blockers	11 (41)	NA	NA
Angiotensin-converting enzyme inhibitors	10 (37)	NA	NA
β-Blockers	12 (44)	NA	NA
Calcium channel blockers	6 (22)	NA	NA
Nitrates	1 (4)	NA	NA

Abbreviation: NA, not applicable.

^a^ Calculated as weight in kilograms divided by height in meters squared.

^b^ Measured in only 15 HCs.

^c^ Values derived from the parent clinical trial.^28^

### Exercise Performance

Patients with HFpEF had severely reduced peak VO_2_ compared with HCs (Table 2).^9,47^ This was evident despite similar peak exercise respiratory exchange ratio in HFpEF vs HC, which was 1.08 or more in both groups, indicating exhaustive exercise effort. There was nonsignificantly lower peak heart rate in patients with HFpEF vs HC. Six-minute walk distance was also significantly reduced in HFpEF compared with HC. Both SPPB chair stand and walk (4 m) times were higher in patients with HFpEF compared with HC, resulting in overall lower SPPB scores in patients with HFpEF. Left leg strength was significantly reduced in patients with HFpEF compared with HCs.

**Table 2.** Cardiopulmonary and Hemodynamic Responses During Peak Treadmill Exercise and Physical Function Measures

Measure	Raw data, mean (SD)			Adjusted data, mean (SE)^a^		
	Patients with HFpEF	HCs	*P* value	Patients with HFpEF	HCs	*P* value
Peak VO_2_, mL/kg/min	14.8 (3.0)	26.0 (6.6)	<.001	15.0 (1.0)	25.9 (0.8)	<.001
Peak VO_2_, mL/min	1539 (393)	1923 (643)	.007	1569 (84)	1905 (65)	.002
Peak VCO_2_, mL/min	1665 (433)	2162 (832)	.005	1703 (105)	2139 (82)	.002
Ventilatory anaerobic threshold, mL/min	1111 (271)	1001 (367)	.91	1022 (57)	988 (44)	.64
Peak respiratory exchange ratio	1.08 (0.07)	1.11 (0.10)	.17	1.08 (0.02)	1.11 (0.01)	.21
Peak heart rate, beats per min	138 (22)	152 (16)	.002	138 (4)	152 (3)	.003
Peak systolic blood pressure, mm Hg	190 (22)	176 (17)	.003	190 (4)	176 (3)	.002
Peak diastolic blood pressure, mm Hg	81 (10)	79 (11)	.68	81 (2)	79 (1)	.50
6-min Walk distance, m	373 (61)	546 (94)	<.001	375 (16)	545 (12)	<.001
SPPB 4-m walk time, s	5.1 (1.0)	3.7 (0.9)	<.001	5.1 (0.2)	3.7 (0.1)	<.001
SPPB chair time, s	14.1 (4.0)	9.6 (2.0)	<.001	14.1 (0.6)	9.6 (0.4)	<.001
SPPB total score, units	9.9 (1.6)	11.6 (1.1)	<.001	9.9 (0.2)	11.6 (0.2)	<.001
Right leg strength, N · m	87.0 (27.2)	101.5 (37.3)	.14	87.9 (7.2)	100.6 (5.0)	.15
Left leg strength, N · m	79.9 (21.3)	107.7 (35.2)	.006	81.9 (7.1)	105.6 (4.4)	.007

Abbreviations: HFpEF, heart failure with preserved ejection fraction; HC, healthy control; peak VCO_2_, peak carbon dioxide output; peak VO_2_, peak exercise oxygen consumption; SPPB, Short Physical Performance Battery.

^a^ Adjusted for sex.

### Skeletal Muscle Bioenergetic Characteristics

A representative trace depicting the high-resolution respirometry protocol used for this project is shown in eFigure 1 in Supplement 1. Comparisons of bioenergetic measures between patients with HFpEF and HCs are shown in Table 3. Across complex I respiration, complexes I and II respiration, and maximal capacity, the mean oxygen consumption rates per mg muscle were significantly reduced in patients with HFpEF compared with HCs. When the data were adjusted for sex, age, and BMI, individually and together, differences remained statistically significant.

**Table 3.** Skeletal Muscle Mitochondrial Respirometry

Parameter	Oxygen consumption rate, mean (SE), pmol/s^−1^/mg muscle		*P* value^a^	*P* value^b^	*P* value^c^	*P* value^d^	*P* value^e^
	Patients with HFpEF (n = 27)	HCs (n = 45)					
Complex I respiration	10.7 (3.8)	28.2 (8.0)	<.001	<.001	<.001	<.001	<.001
Complexes I and II respiration	15.9 (5.4)	40.1 (11.8)	<.001	<.001	<.001	<.001	<.001
Maximal capacity	24.4 (9.4)	61.4 (13.7)	<.001	<.001	<.001	<.001	<.001

Abbreviations: HFpEF, heart failure with preserved ejection fraction; HC, healthy control.

^a^ Unadjusted.

^b^ Adjusted for sex.

^c^ Adjusted for age.

^d^ Adjusted for body mass index.

^e^ Adjusted for sex, age, and body mass index.

### Correlations Between Skeletal Muscle Bioenergetics and Measures of Physical Function

Pearson correlation coefficients were used to examine correlations between skeletal muscle mitochondrial function and measures of physical function across participants with HFpEF and HCs. The results are summarized in eTable 1 in Supplement 1. Both absolute and relative peak VO_2_ (per kg weight) were significantly positively correlated with complex I respiration (*R* = 0.70; *P* < .001), complexes I and II respiration (*R* = 0.69; *P* < .001), and maximal capacity (*R* = 0.69; *P* < .001) (Figure, A). Similarly, 6-minute walk distance (*R* = 0.69; *P* < .001) and leg strength (*R* = 0.41; *P* < .001) had positive correlations with skeletal muscle respiration (Figure, B and C). SPPB scores were positively correlated with skeletal muscle fiber respiration (*R* = 0.46; *P* < .001), while its individual components, gait time and chair stands, showed negative correlations (Figure, D; eTable 1 in Supplement 1). Sensitivity analyses were conducted to assess whether the correlation between skeletal muscle mitochondrial function and peak VO_2_ differed between the patients with HFpEF and HCs using both stratified correlation analysis (eTable 2 and eFigure 2 in Supplement 1) and regression analysis with an interaction term. Results of the sensitivity analyses indicate similar correlations between patients with HFpEF (*R* = 0.28; *P* = .15) and HCs (*R* = 0.28; *P* = .06) for peak VO_2_ and maximal capacity, respectively, and all tests for interaction were nonsignificant.

**Figure.**
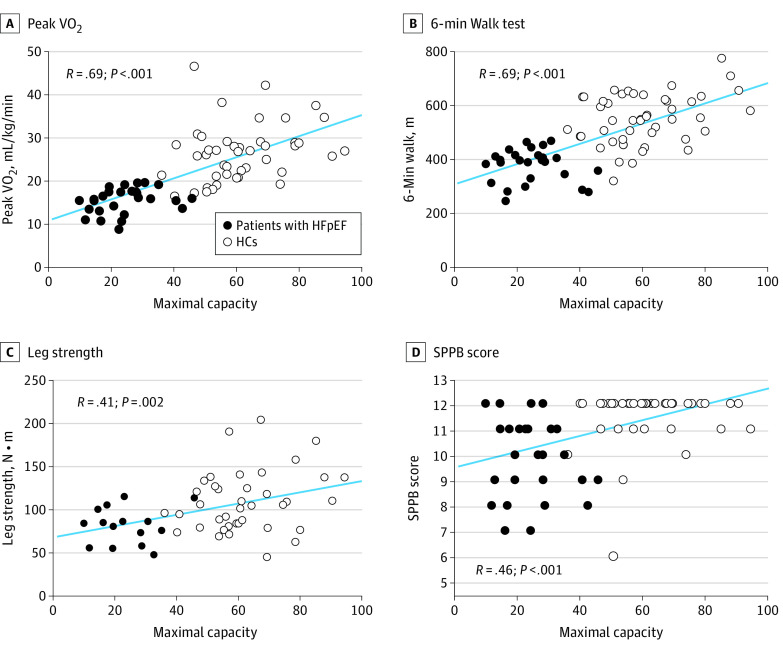
Associations of Maximal Capacity With Exercise Capacity and Physical Ability The blue line indicates the simple linear regression line. HC indicates healthy control; HFpEF, heart failure with preserved ejection fraction; peak VO_2_, peak exercise oxygen consumption; SPPB, Short Physical Performance Battery.

## Discussion

This study using respirometry of skeletal muscle fiber bundles obtained from biopsy in patients with HFpEF compared with age-matched HCs, to our knowledge, provides the first direct evidence indicating that cellular-level mitochondrial dysfunction underlies skeletal muscle metabolic abnormalities in those with HFpEF and is associated with multiple objective measures of severe exercise intolerance, including reduced peak VO_2_, 6-minute walk distance, SPPB, and leg strength. These mitochondrial abnormalities represent potential therapeutic targets, as the function of these organelles is linked to many common disease processes and potentially modifiable by both pharmacological interventions as well as behavioral interventions, such as diet and exercise.^48^

Patients with chronic HFpEF have severe exercise intolerance that is associated with impaired quality of life.^7,9,15^ To date, the mechanistic underpinnings of HFpEF exercise intolerance remain incompletely understood, thus hampering efforts to develop effective interventions. Multiple lines of evidence indicate that in addition to cardiac factors, noncardiac peripheral factors, including abnormal skeletal muscle metabolism, contribute to the severe exercise intolerance in patients with HFpEF.^9,12,15,49,50,51,52^ We have previously reported that mitochondrial content and oxidative capacity are significantly reduced in skeletal muscle biopsy samples from patients with HFpEF.^19^ Furthermore, we reported that expression of Mfn2, a key mediator of mitochondrial fusion, is similarly decreased. Notably, these mitochondrial impairments were associated with measures of exercise intolerance, specifically peak VO_2_ and 6-minute walk distance. However, these previous studies examining mitochondria in patients with HFpEF were limited due to the reliance on stored frozen tissues samples, which prevented analyses of mitochondrial function by respirometry, a direct and precise approach for assessing mitochondrial function.

In this study, we used high-resolution respirometric profiling of permeabilized skeletal muscle fiber bundles to provide, to our knowledge, the first direct analysis of mitochondrial function in patients with HFpEF. The major new finding is that compared with age-matched HCs, patients with HFpEF had lower mitochondrial respiration across measures of oxidative phosphorylation capacity of the nicotinamide adenine dinucleotide plus hydrogen (NADH) pathway through complex I respiration, convergent NADH and succinate (NS) pathways through complexes I and II respiration, and electron transfer capacity of the convergent NS pathway. Multiple lines of evidence indicate that excess adipose tissue is associated with impaired mitochondrial function and reduced mitochondrial density.^53,54^ Given that more than 80% of patients with HFpEF are overweight or obese, twice the rate found in the general older population,^44,45^ we analyzed whether this difference could contribute to impaired skeletal muscle function. Adjusting for BMI did not affect the differences in mitochondrial respiration we observed between participants with HFpEF and HCs. Thus, our results indicate that factors unrelated to obesity also contribute to skeletal muscle mitochondrial dysfunction in patients with HFpEF. This is consistent with findings from an animal model of HFpEF, which showed reduced skeletal muscle mitochondrial density compared with controls, despite no difference in body mass.^20^ Similarly, adjusting for age did not account for differences in mitochondrial respiration.

Among healthy persons, skeletal muscle mitochondrial function has been shown to be directly related to physical function and exercise capacity, supporting our observation that mitochondrial abnormalities in patients with HFpEF are associated with their severely impaired physical function.^55,56^ Coen et al^57^ reported that the respiratory capacity of muscle fibers and maximal phosphorylation capacity are associated with peak VO_2_ and walk speed in older adults. Notably, the average peak VO_2_ in that study was 22.0 mL·min^−1^·kg^−1^, while the average peak VO_2_ in our HFpEF cohort was 14.8 mL·min^−1^·kg^−1^, in line with differences associated with disease.

Mitochondrial abnormalities are emerging as promising therapeutic targets for a number of common disorders, particularly those associated with aging, such as HFpEF.^48^ Notably, mitochondrial function is modifiable and responsive to interventions. The present data provide the foundation for future studies to examine interventions, such as exercise training, which has been shown to positively affect skeletal muscle mitochondria function.^58,59^ The present data suggest the possibility that alterations in mitochondrial function may underlie our previous observation that the increase in arteriovenous oxygen difference, which accounts for 90% of the increase in peak VO_2_ in patients with HFpEF following exercise training, is primarily due to noncardiac factors, particularly skeletal muscle function,^14,15,60^ since arterial stiffness and conduit arterial endothelial dysfunction do not appear to improve with exercise training in HFpEF.^51^ This concept should be confirmed in future exercise intervention studies using bioenergetic profiling strategies similar to those used in the present study. Pharmacological interventions targeting mitochondrial abnormalities are also in development. For example, Szeto-Schiller peptides have been shown to target mitochondrial dysfunction in myocytes and are being tested in clinical trials.^61^ Additional potentially therapeutic molecules targeting mitochondria include coenzyme Q10, MitoQ, mitochondrial division inhibitor 1, and nicotinamide mononucleotide.^48^ Notably, previous clinical trials have been focused on effects on cardiac function in patients with HFpEF. The present data suggest that clinical trials targeting mitochondrial abnormalities in HFpEF may have beneficial effects on skeletal muscle metabolism and exercise performance.

### Strengths and Limitations

A primary strength of this article is the use of high-resolution respirometry to directly assess mitochondrial function in freshly isolated permeabilized skeletal muscle fiber bundles. These precise ex vivo measurements have significantly advanced our understanding of human muscle metabolism. To our knowledge, the study presented here is the largest to use these assays in patients with HFpEF. Other strengths include an age-matched HC group and the multiple measures of physical function and exercise capacity, including peak VO_2_, 6-minute walk distance, SPPB, and leg strength, to determine their association with the mitochondrial abnormalities.

This study has limitations. Patients with HFpEF compared with HC participants were well matched for age and sex distribution but had higher BMI. While this reflects the nature of HFpEF, since more than 80% of patients with HFpEF are overweight or obese, twice the rate of the general population, it creates uncertainty regarding independent effects, since excess adiposity may affect skeletal muscle mitochondria^53,59,62^; hence, the potential associations of obesity with skeletal muscle mitochondria should be considered in the interpretation of these results. However, we included models adjusting for BMI individually and in conjunction with age and sex in our analyses and found that differences between participants with HFpEF and HCs were largely unaffected. Another potential difference that could affect our readouts of skeletal muscle mitochondrial function is the relative abundance of type 1 muscle fibers, which have a distinct metabolic phenotype. Our team has previously reported that the relative abundance of type 1 fibers is lower in the skeletal muscle of participants with HFpEF^18^; however, comparative data reporting on the abundance of type 1 fibers are not available in this study.

Several studies in both animal models and humans have reported that physical activity and sedentary behavior are related to skeletal muscle mitochondrial function.^63,64^ A limitation of this work is that differences in physical activity and sedentary behavior, which have been reported in patients with HFpEF,^65,66^ were not measured in this study. While we cannot definitely decipher whether skeletal muscle mitochondrial dysfunction is a cause or consequence of differences in physical activity in HFpEF compared with HCs, it should be noted that reduced physical function (such as pVO_2_) in HFpEF is not merely due to sedentary behavior. This concept is supported by multiple lines of evidence for skeletal myopathy in HFpEF, as previously reviewed by our team.^60^ Similarly, the data presented in this article serve to highlight the presence of severe skeletal muscle mitochondrial dysfunction in patients with HFpEF and reports on the correlations of these measures with multiple measures of physical ability and fitness. However, the causal relationship between mitochondrial dysfunction and exercise intolerance in patients with HFpEF remains to be determined.

## Conclusions

In this study, older patients with HFpEF showed marked abnormalities in mitochondrial function that were significantly associated with their reduced exercise capacity and muscle strength. These results provide new insights into potential novel therapeutic targets.

## Notes

Supplement 1. **eTable 1.** Correlation Between Skeletal Muscle Respiration and Physical Function **eTable 2.** Correlation Between Skeletal Muscle Respiration and Peak VO_2_ by HFpEF Status **eFigure 1.** Representative High-Resolution Respirometry Trace **eFigure 2.** Association Between Skeletal Muscle Respiration and Peak VO_2_ in HFpEF and Healthy Controls

Supplement 2. Data Sharing Statement
