# Supplementary material for: Skeletal Muscle Mitochondrial Respiration and Exercise Intolerance in Patients With Heart Failure With Preserved Ejection Fraction
Source: JAMA Cardiol. 2023 May 10;8(6):575–84. doi: 10.1001/jamacardio.2023.0957 (PMC10173105; doi:10.1001/jamacardio.2023.0957)
Supplement: Supplement 1. — eTable 1. Correlation Between Skeletal Muscle Respiration and Physical Function eTable 2. Correlation Between Skeletal Muscle Respiration and Peak VO2 by HFpEF Status eFigure 1. Representative High-Resolution Respirometry Trace eFigure 2. Association Between Skeletal Muscle Respiration and Peak VO2 in HFpEF and Healthy Controls [file jamacardiol-e230957-s001.pdf]

Supplemental Online Content

Scandalis L, Kitzman DW, Nicklas BJ, et al. Skeletal muscle mitochondrial respiration and exercise intolerance in patients with heart failure with preserved ejection fraction. *JAMA Cardiol*. Published online May 10, 2023. doi:10.1001/jamacardio.2023.0957

eTable 1. Correlation Between Skeletal Muscle Respiration and Physical Function
eTable 2. Correlation Between Skeletal Muscle Respiration and Peak VO_2 by HFpEF Status
eFigure 1. Representative High-Resolution Respirometry Trace
eFigure 2. Association Between Skeletal Muscle Respiration and Peak VO_2 in HFpEF and Healthy Controls

This supplementary material has been provided by the authors to give readers additional information about their work.

© 2023 Scandalis L et al. *JAMA Cardiology*.

eTable 1. Correlation Between Skeletal Muscle Respiration and Physical Function

Physical Function Measurements	CXI, N_P		CXI+CXII, NS_P		MAX ETS, NS_E	
	r	*p*-value	*r*	*p*-value	*r*	*p*-value
Peak VO_2, mL/kg/min	0.70	<.001	0.69	<.001	0.69	<.001
Peak VO_2, mL/min	0.43	<.001	0.47	<.001	0.41	<.001
6-minute walk distance	0.69	<.001	0.69	<.001	0.69	<.001
SPPB total	0.44	<.001	0.44	<.001	0.46	<.001
SPPB gait speed	-0.51	<.001	-0.52	<.001	-0.53	<.001
SPPB chair time	-0.53	<.001	-0.52	<.001	-0.55	<.001
Leg Strength – right	0.24	.07	0.26	.05	0.21	.11
Leg Strength - left	0.42	.002	0.44	<.001	0.41	.002

Pearson correlation coefficients and p-values for each association are shown.

CXI, Np, NADH-linked pathway through mitochondrial Complex I; CXI+CXII, NSp, NADH/Succinate combined pathway through Complexes I and II; MAX ETS, NS_E, maximum electron transport in the NADH/Succinate ET state; r, Pearson correlation coefficient; Peak VO_2, peak exercise oxygen consumption; SPPB, Short physical performance battery.

© 2023 Scandalis L et al. *JAMA Cardiology*.

eTable 2. Correlation Between Skeletal Muscle Respiration and Peak VO_2 by HFpEF Status

	**Peak VO_2, mL/kg/min**			
	**HFpEF (N=27)**		**HC (N=45)**	
	R	p-value	r	p-value
**CXI, N_P**	0.45	.0194	0.26	.0787
**CXI+CXII, NS_P**	0.47	.0145	0.31	.0393
**MAX ETS, NS_E**	0.28	.1508	0.28	.0642

© 2023 Scandalis L et al. *JAMA Cardiology*.

eFigure 1. Representative High-Resolution Respirometry Trace

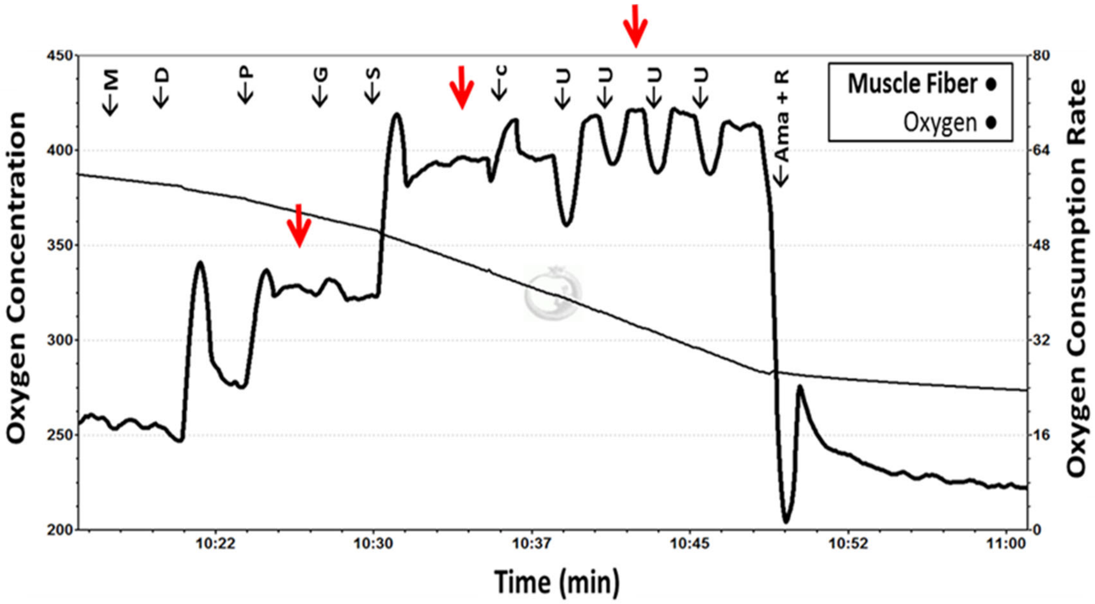

Red arrows (from left to right) depict respiratory control states for OXPHOS capacity of the NADH pathway through Complex I (complex I, NP), the convergent NADH- and succinate pathway through Complex I and Complex II (complex I+complex II, NS_P), and the maximal electron transfer capacity of the systems-pathway (maximal capacity, NS_E).

© 2023 Scandalis L et al. *JAMA Cardiology*.

eFigure 2. Association Between Skeletal Muscle Respiration and Peak VO_2 in HFpEF and Healthy Controls

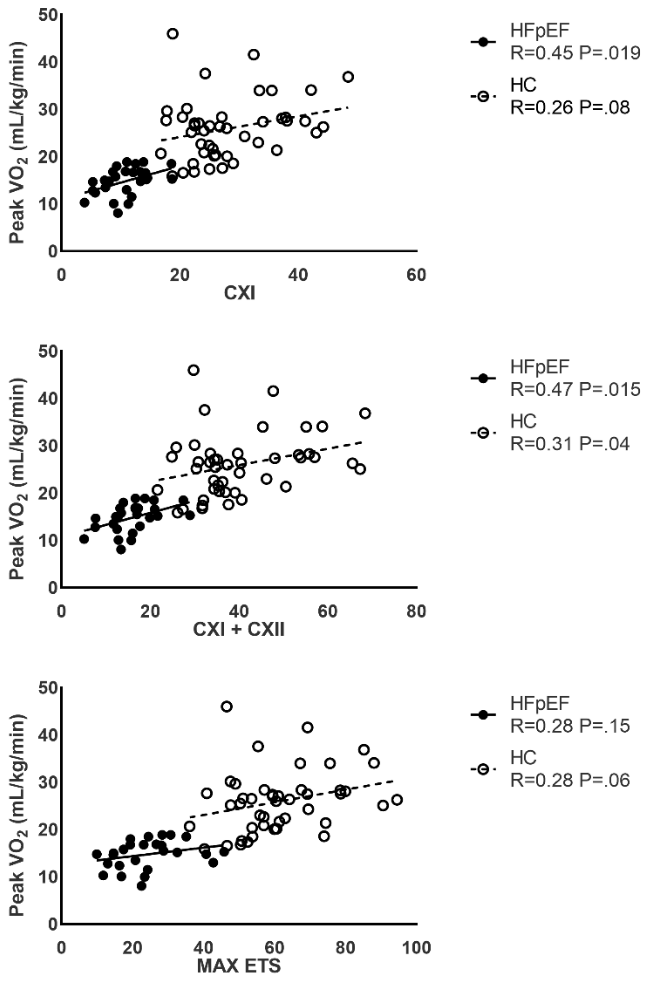

© 2023 Scandalis L et al. *JAMA Cardiology*.
