# Supplementary material for: Skeletal Muscle Mitochondrial Respiration and Exercise Intolerance in Patients With Heart Failure With Preserved Ejection Fraction
Source: JAMA Cardiol. 2023 May 10;8(6):575–84. doi: 10.1001/jamacardio.2023.0957 (PMC10173105; doi:10.1001/jamacardio.2023.0957)
Supplement: Supplement 2. — Data Sharing Statement [file jamacardiol-e230957-s002.pdf]

Data Sharing Statement

Scandalis. Skeletal Muscle Mitochondrial Respiration and Exercise Intolerance in Patients With Heart Failure With Preserved Ejection Fraction. *JAMA Cardiol*. Published May 10, 2023. doi:10.1001/jamacardio.2023.0957

Data
Data available: Yes
Data types: Deidentified participant data
How to access data: ajmolina@health.ucsd.edu
When available: With publication

Supporting Documents
Document types: None

Additional Information
Who can access the data: Researchers whose proposed use of the data has been approved
Types of analyses: Any purpose
Mechanisms of data availability: With investigator support
Any additional restrictions: none
